# Ethyl 7-methyl-2-phenyl­pyrazolo­[1,5-*a*]pyrimidine-5-carboxyl­ate

**DOI:** 10.1107/S1600536813009902

**Published:** 2013-04-17

**Authors:** Ibtissam Bassoude, Sabine Berteina-Raboin, El Mokhtar Essassi, Gérald Guillaumet, Lahcen El Ammari

**Affiliations:** a Laboratoire de Chimie Organique Hétérocyclique URAC21, Pôle de Compétences Pharmacochimie, Université Mohammed V-Agdal, Avenue Ibn Battouta, BP 1014, Rabat, Morocco; b Institut de Chimie Organique et Analytique, Université d’Orléans, UMR CNRS 6005, BP 6759, 45067 Orléans Cedex 2, France; c Institute of Nanomaterials and Nanotechnology, MASCIR, Rabat, Morocco; d Laboratoire de Chimie du Solide Appliquée, Université Mohammed V-Agdal, Faculté des Sciences, Avenue Ibn Battouta, BP 1014, Rabat, Morocco

## Abstract

The fused pyrazole and pyrimidine rings in the title compound, C_16_H_15_N_3_O_2_, are almost coplanar, being inclined to one another by 1.31 (12)°. The mean plane of this fused ring system is nearly coplanar with the phenyl ring, as indicated by the dihedral angle between their planes of 1.31 (12)°. The fused-ring system and the phenyl ring are nearly coplanar, as indicated by the dihedral angle of 1.27 (10)°. In the crystal, mol­ecules form inversion dimers *via* pairs of C—H⋯O hydrogen bonds. C—H⋯N inter­actions connect the dimers into a three-dimensional network. In addition, π–π contacts are observed, with centroid–centroid distances of 3.426 (2) Å.

## Related literature

For pharmacological and biochemical properties of pyrazolo­[1,5-*a*]pyrimidine derivatives, see: Selleri *et al.* (2005 ▶); Almansa *et al.* (2001 ▶); Suzuki *et al.* (2001 ▶); Chen *et al.* (2004 ▶). For related structures, see: Chimichi *et al.* (1992 ▶).
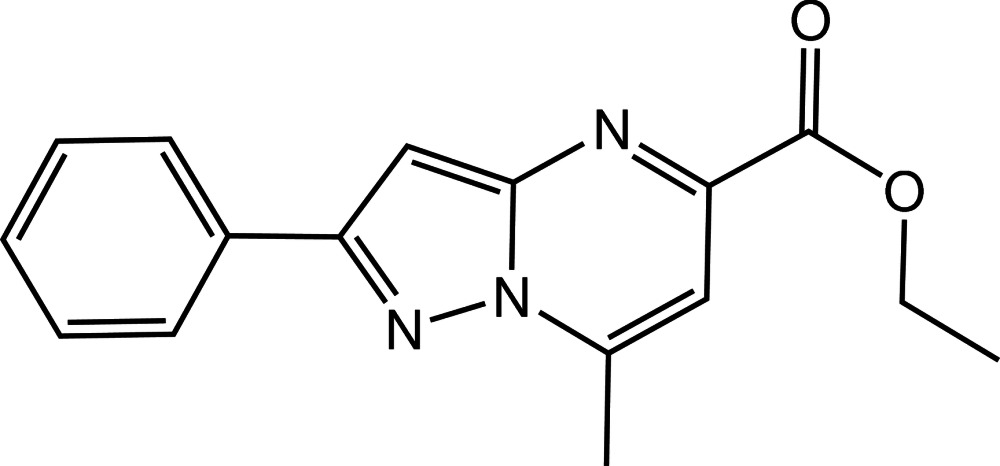


## Experimental

### 

#### Crystal data

C_16_H_15_N_3_O_2_
*M* *_r_* = 281.31
Orthorhombic, 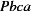

*a* = 8.0542 (8) Å
*b* = 16.4104 (19) Å
*c* = 21.635 (2) Å
*V* = 2859.5 (5) Å^3^
*Z* = 8
Mo *K*α radiation
μ = 0.09 mm^−1^
*T* = 296 K
0.41 × 0.32 × 0.21 mm

#### Data collection

Bruker X8 APEXII area-detector diffractometer
12894 measured reflections
2783 independent reflections
1919 reflections with *I* > 2σ(*I*)
*R*_int_ = 0.033

#### Refinement

*R*[*F* ^2^ > 2σ(*F* ^2^)] = 0.052
*wR*(*F* ^2^) = 0.170
*S* = 1.04
2783 reflections
190 parameters
H-atom parameters constrained
Δρ_max_ = 0.35 e Å^−3^
Δρ_min_ = −0.36 e Å^−3^

### 

Data collection: *APEX2* (Bruker, 2009 ▶); cell refinement: *SAINT* (Bruker, 2009 ▶); data reduction: *SAINT*; program(s) used to solve structure: *SHELXS97* (Sheldrick, 2008 ▶); program(s) used to refine structure: *SHELXL97* (Sheldrick, 2008 ▶); molecular graphics: *ORTEP-3 for Windows* (Farrugia, 2012 ▶); software used to prepare material for publication: *PLATON* (Spek, 2009 ▶) and *publCIF* (Westrip, 2010 ▶).

## Supplementary Material

Crystal structure: contains datablock(s) I, global. DOI: 10.1107/S1600536813009902/rz5057sup1.cif

Structure factors: contains datablock(s) I. DOI: 10.1107/S1600536813009902/rz5057Isup2.hkl

Supplementary material file. DOI: 10.1107/S1600536813009902/rz5057Isup3.cml

Additional supplementary materials:  crystallographic information; 3D view; checkCIF report

## Figures and Tables

**Table 1:** Hydrogen-bond geometry (Å, °)

*D*—H⋯*A*	*D*—H	H⋯*A*	*D*⋯*A*	*D*—H⋯*A*
C12—H12⋯O1^i^	0.93	2.36	3.258 (3)	161
C6—H6⋯N3^ii^	0.93	2.62	3.507 (3)	161

Symmetry codes: (i) 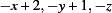; (ii) 
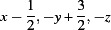
.

**Fig. 1.**
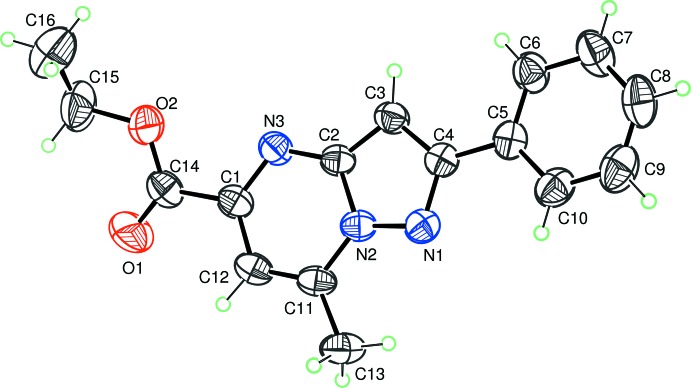
The molecular structure of the title compound with displacement ellipsoids drawn at the 50% probability level. H atoms are represented as small circles.

**Fig. 2.**
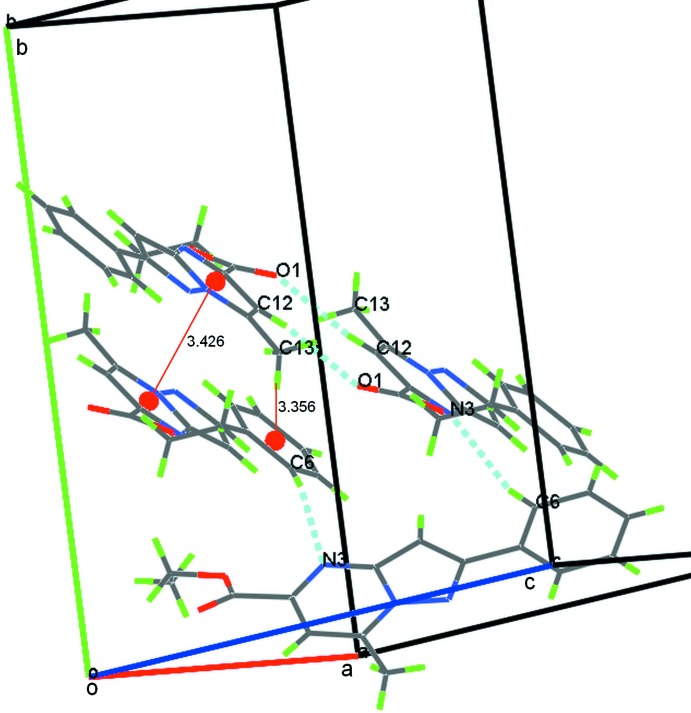
Crystal packing of the title compound showing C12–H12···O1 and C6–H6···N3 hydrogen bonds as blue dashed lines, with C—H···π interactions and a π—π contact (red lines). The red spheres represent the centroids of the C5–C10 and N2/N3/C1/C2/C11/C12 rings and their symmetry partners.

## supplementary crystallographic information

### Comment

Pyrazolo[1,5-*a*]pyrimidines have attracted considerable interest because of their biological activity. For instance, they are known for their potent utility as selective peripheral benzodiazepine receptor ligands (Selleri *et al.*, 2005), COX-2 selective inhibitors (Almansa *et al.*, 2001), HMG-CoA reductase inhibitors (Suzuki *et al.*, 2001) and CRF1 antagonists (Chen *et al.*, 2004).

The condensation of 5-amino-3-arylpyrazoles with ethyl 2,4-dioxopentanoate leads to the title compound ethyl-7-methyl-2-phenylpyrazolo[1,5-*a*]pyrimidine- 5-carboxylate and its isomeric ethyl 5-methyl-2-phenylpyrazolo[1,5-*a*]pyrimidine-7-carboxylate (Chimichi *et al.*, 1992).

The crystal structure of the title compound is built up from two fused five and six-membered rings (N1/N2/C2–C4 and N2/N3/C1/C2/C11/C12) linked to a methyl, a phenyl (C5–C10) and to an ethylcarboxylate group (C14/O1/O2/C15/C16) as shown in Fig. 1. The pyrazole and pyrimidine rings are almost planar with a maximum deviation for atom C6 of 0.002 (2) Å and 0.004 (2) Å, respectively. The mean plane through the two fused rings is slightly folded around the common edge as indicated by the dihedral angle between them of 1.31 (12)°. The dihedral angle between the phenyl ring and the fused-ring system is 1.27 (10)°.

In the crystal structure C12–H12···O1 hydrogen bonds form inversion dimers. Intermolecular C6—H6···N3 interactions connect the dimers into a three dimensional network. In addition, the molecules are connected by π–π contacts, with centroid–centroid distances of 3.426 (2) Å.

### Experimental

A solution of ethyl 2,4-dioxopentanoate (1.64 g, 10.4 mmol) and 5-amino-3-phenylpyrazole (1.5 g, 9.4 mmol) in 10 ml of EtOH was heated to reflux for 30 min. After evaporation of solvent under reduced pressure, the residue was purified on silica gel by column chromatography using a 8:2 (*v*/*v*) mixture of petroleum ether and ethyl acetate as eluent. Ethyl-7-methyl-2-phenylpyrazolo[1,5-*a*]pyrimidine-5-carboxylate was recrystallized from cyclohexane to give colourless crystals.

### Refinement

All H atoms could be located in a difference Fourier map and treated as riding with C—H = 0.93 Å (aromatic), C—H = 0.97 Å (methylene) and C—H = 0.96 Å (methyl), and with *U*_iso_(H) = 1.2 *U*_eq_(aromatic, methylene) or *U*_iso_(H) = 1.5 *U*_eq_ (methyl).

### Crystal data

**Table:**

C_16_H_15_N_3_O_2_	*F*(000) = 1184
*M**_r_* = 281.31	*D*_x_ = 1.307 Mg m^−^^3^
Orthorhombic, *P**b**c**a*	Mo *K*α radiation, λ = 0.71073 Å
Hall symbol: -p 2ac 2ab	Cell parameters from 2783 reflections
*a* = 8.0542 (8) Å	θ = 2.5–26.0°
*b* = 16.4104 (19) Å	µ = 0.09 mm^−^^1^
*c* = 21.635 (2) Å	*T* = 296 K
*V* = 2859.5 (5) Å^3^	Block, colourless
*Z* = 8	0.41 × 0.32 × 0.21 mm

### Data collection

**Table:**

Bruker X8 APEXII area-detector diffractometer	1919 reflections with *I* > 2σ(*I*)
Radiation source: fine-focus sealed tube	*R*_int_ = 0.033
Graphite monochromator	θ_max_ = 26.0°, θ_min_ = 2.5°
φ and ω scans	*h* = −9→9
12894 measured reflections	*k* = −20→19
2783 independent reflections	*l* = −25→26

### Refinement

**Table:**

Refinement on *F*^2^	Primary atom site location: structure-invariant direct methods
Least-squares matrix: full	Secondary atom site location: difference Fourier map
*R*[*F*^2^ > 2σ(*F*^2^)] = 0.052	Hydrogen site location: difference Fourier map
*wR*(*F*^2^) = 0.170	H-atom parameters constrained
*S* = 1.04	*w* = 1/[σ^2^(*F*_o_^2^) + (0.0849*P*)^2^ + 1.249*P*] where *P* = (*F*_o_^2^ + 2*F*_c_^2^)/3
2783 reflections	(Δ/σ)_max_ < 0.001
190 parameters	Δρ_max_ = 0.35 e Å^−^^3^
0 restraints	Δρ_min_ = −0.36 e Å^−^^3^

### Special details

**Table:**

Geometry. All e.s.d.'s (except the e.s.d. in the dihedral angle between two l.s. planes) are estimated using the full covariance matrix. The cell e.s.d.'s are taken into account individually in the estimation of e.s.d.'s in distances, angles and torsion angles; correlations between e.s.d.'s in cell parameters are only used when they are defined by crystal symmetry. An approximate (isotropic) treatment of cell e.s.d.'s is used for estimating e.s.d.'s involving l.s. planes.
Refinement. Refinement of *F*^2^ against all reflections. The weighted *R*-factor *wR* and goodness of fit *S* are based on *F*^2^, conventional *R*-factors *R* are based on *F*, with *F* set to zero for negative *F*^2^. The threshold expression of *F*^2^ > σ(*F*^2^) is used only for calculating *R*-factors(gt) *etc*. and is not relevant to the choice of reflections for refinement. *R*-factors based on *F*^2^ are statistically about twice as large as those based on *F*, and *R*- factors based on all data will be even larger.

### Fractional atomic coordinates and isotropic or equivalent isotropic displacement parameters (Å^2^)

**Table:**

	*x*	*y*	*z*	*U*_iso_*/*U*_eq_	
C1	0.7015 (3)	0.59846 (13)	−0.00919 (11)	0.0423 (5)	
C2	0.4533 (2)	0.62909 (13)	0.03355 (10)	0.0392 (5)	
C3	0.2988 (3)	0.66217 (13)	0.04562 (11)	0.0431 (5)	
H3	0.2464	0.7041	0.0243	0.052*	
C4	0.2383 (3)	0.61938 (13)	0.09644 (10)	0.0404 (5)	
C5	0.0780 (3)	0.63145 (14)	0.12822 (10)	0.0426 (5)	
C6	−0.0290 (3)	0.69261 (15)	0.10875 (12)	0.0531 (6)	
H6	0.0002	0.7255	0.0755	0.064*	
C7	−0.1796 (3)	0.70489 (17)	0.13873 (14)	0.0660 (8)	
H7	−0.2511	0.7456	0.1252	0.079*	
C8	−0.2234 (4)	0.65694 (18)	0.18851 (14)	0.0689 (8)	
H8	−0.3243	0.6650	0.2085	0.083*	
C9	−0.1158 (3)	0.5968 (2)	0.20843 (13)	0.0681 (8)	
H9	−0.1438	0.5649	0.2424	0.082*	
C10	0.0323 (3)	0.58363 (17)	0.17833 (12)	0.0557 (7)	
H10	0.1025	0.5422	0.1917	0.067*	
C11	0.6159 (3)	0.52087 (13)	0.07911 (10)	0.0423 (5)	
C12	0.7310 (3)	0.53683 (14)	0.03464 (11)	0.0455 (6)	
H12	0.8291	0.5070	0.0332	0.055*	
C13	0.6281 (3)	0.45638 (16)	0.12736 (12)	0.0584 (7)	
H13A	0.5308	0.4578	0.1530	0.088*	
H13B	0.7247	0.4658	0.1523	0.088*	
H13C	0.6365	0.4040	0.1079	0.088*	
C14	0.8285 (3)	0.61382 (15)	−0.05871 (12)	0.0493 (6)	
C15	0.8726 (4)	0.6665 (2)	−0.15930 (14)	0.0784 (9)	
H15A	0.9632	0.6276	−0.1601	0.094*	
H15B	0.9191	0.7208	−0.1558	0.094*	
C16	0.7733 (6)	0.6597 (3)	−0.21607 (16)	0.1145 (15)	
H16A	0.8427	0.6700	−0.2513	0.172*	
H16B	0.6848	0.6989	−0.2150	0.172*	
H16C	0.7276	0.6058	−0.2190	0.172*	
N1	0.3450 (2)	0.56159 (11)	0.11673 (9)	0.0435 (5)	
N2	0.4760 (2)	0.56790 (11)	0.07779 (8)	0.0395 (5)	
N3	0.5670 (2)	0.64421 (11)	−0.01033 (9)	0.0424 (5)	
O1	0.9709 (2)	0.59496 (15)	−0.05385 (11)	0.0868 (7)	
O2	0.76394 (19)	0.65002 (11)	−0.10746 (8)	0.0575 (5)	

### Atomic displacement parameters (Å^2^)

**Table:**

	*U*^11^	*U*^22^	*U*^33^	*U*^12^	*U*^13^	*U*^23^
C1	0.0374 (11)	0.0394 (12)	0.0500 (14)	−0.0004 (9)	−0.0033 (9)	−0.0011 (11)
C2	0.0402 (11)	0.0342 (11)	0.0432 (13)	−0.0002 (9)	−0.0029 (9)	0.0031 (10)
C3	0.0424 (11)	0.0386 (12)	0.0484 (14)	0.0045 (9)	0.0018 (10)	0.0049 (10)
C4	0.0398 (11)	0.0401 (11)	0.0411 (13)	−0.0031 (9)	−0.0021 (9)	−0.0020 (10)
C5	0.0410 (11)	0.0439 (12)	0.0429 (13)	−0.0096 (9)	0.0017 (9)	−0.0050 (11)
C6	0.0491 (13)	0.0494 (14)	0.0607 (16)	−0.0019 (11)	0.0115 (11)	0.0015 (12)
C7	0.0535 (14)	0.0578 (16)	0.087 (2)	0.0039 (12)	0.0200 (14)	−0.0032 (16)
C8	0.0566 (16)	0.074 (2)	0.076 (2)	−0.0108 (14)	0.0254 (14)	−0.0131 (17)
C9	0.0625 (16)	0.083 (2)	0.0586 (18)	−0.0192 (15)	0.0127 (13)	0.0060 (15)
C10	0.0517 (13)	0.0662 (16)	0.0492 (15)	−0.0084 (12)	−0.0017 (11)	0.0072 (13)
C11	0.0392 (11)	0.0382 (12)	0.0496 (14)	−0.0004 (9)	−0.0137 (9)	0.0017 (11)
C12	0.0359 (10)	0.0436 (13)	0.0570 (15)	0.0039 (9)	−0.0077 (10)	−0.0002 (12)
C13	0.0522 (14)	0.0578 (15)	0.0650 (17)	0.0032 (12)	−0.0134 (12)	0.0171 (13)
C14	0.0387 (12)	0.0475 (13)	0.0618 (16)	0.0016 (10)	0.0021 (10)	−0.0024 (12)
C15	0.0669 (17)	0.101 (2)	0.068 (2)	−0.0081 (17)	0.0267 (15)	0.0040 (18)
C16	0.124 (3)	0.146 (4)	0.073 (3)	0.013 (3)	0.020 (2)	0.028 (3)
N1	0.0415 (9)	0.0463 (11)	0.0428 (11)	−0.0036 (8)	−0.0023 (8)	0.0023 (9)
N2	0.0384 (9)	0.0375 (10)	0.0425 (11)	−0.0020 (7)	−0.0056 (8)	0.0024 (8)
N3	0.0388 (9)	0.0402 (10)	0.0482 (11)	0.0021 (8)	0.0031 (8)	0.0030 (9)
O1	0.0407 (10)	0.1158 (18)	0.1039 (17)	0.0204 (10)	0.0123 (10)	0.0295 (14)
O2	0.0442 (9)	0.0723 (12)	0.0561 (11)	0.0020 (8)	0.0098 (8)	0.0099 (10)

### Geometric parameters (Å, º)

**Table:**

C1—N3	1.318 (3)	C9—H9	0.9300
C1—C12	1.407 (3)	C10—H10	0.9300
C1—C14	1.503 (3)	C11—C12	1.361 (3)
C2—N3	1.342 (3)	C11—N2	1.366 (3)
C2—C3	1.383 (3)	C11—C13	1.490 (3)
C2—N2	1.399 (3)	C12—H12	0.9300
C3—C4	1.393 (3)	C13—H13A	0.9600
C3—H3	0.9300	C13—H13B	0.9600
C4—N1	1.353 (3)	C13—H13C	0.9600
C4—C5	1.476 (3)	C14—O1	1.192 (3)
C5—C10	1.388 (3)	C14—O2	1.318 (3)
C5—C6	1.389 (3)	C15—O2	1.448 (3)
C6—C7	1.390 (3)	C15—C16	1.470 (5)
C6—H6	0.9300	C15—H15A	0.9700
C7—C8	1.380 (4)	C15—H15B	0.9700
C7—H7	0.9300	C16—H16A	0.9600
C8—C9	1.382 (4)	C16—H16B	0.9600
C8—H8	0.9300	C16—H16C	0.9600
C9—C10	1.376 (4)	N1—N2	1.354 (2)
			
N3—C1—C12	124.1 (2)	N2—C11—C13	118.0 (2)
N3—C1—C14	116.8 (2)	C11—C12—C1	120.0 (2)
C12—C1—C14	119.09 (19)	C11—C12—H12	120.0
N3—C2—C3	132.5 (2)	C1—C12—H12	120.0
N3—C2—N2	121.83 (18)	C11—C13—H13A	109.5
C3—C2—N2	105.69 (18)	C11—C13—H13B	109.5
C2—C3—C4	105.43 (19)	H13A—C13—H13B	109.5
C2—C3—H3	127.3	C11—C13—H13C	109.5
C4—C3—H3	127.3	H13A—C13—H13C	109.5
N1—C4—C3	112.77 (19)	H13B—C13—H13C	109.5
N1—C4—C5	119.9 (2)	O1—C14—O2	124.6 (2)
C3—C4—C5	127.3 (2)	O1—C14—C1	123.3 (2)
C10—C5—C6	118.7 (2)	O2—C14—C1	112.16 (18)
C10—C5—C4	121.3 (2)	O2—C15—C16	107.7 (3)
C6—C5—C4	119.9 (2)	O2—C15—H15A	110.2
C5—C6—C7	120.3 (2)	C16—C15—H15A	110.2
C5—C6—H6	119.8	O2—C15—H15B	110.2
C7—C6—H6	119.8	C16—C15—H15B	110.2
C8—C7—C6	120.3 (3)	H15A—C15—H15B	108.5
C8—C7—H7	119.8	C15—C16—H16A	109.5
C6—C7—H7	119.8	C15—C16—H16B	109.5
C7—C8—C9	119.4 (2)	H16A—C16—H16B	109.5
C7—C8—H8	120.3	C15—C16—H16C	109.5
C9—C8—H8	120.3	H16A—C16—H16C	109.5
C10—C9—C8	120.5 (3)	H16B—C16—H16C	109.5
C10—C9—H9	119.7	C4—N1—N2	103.87 (17)
C8—C9—H9	119.7	N1—N2—C11	125.91 (18)
C9—C10—C5	120.7 (3)	N1—N2—C2	112.24 (16)
C9—C10—H10	119.6	C11—N2—C2	121.85 (18)
C5—C10—H10	119.6	C1—N3—C2	116.24 (19)
C12—C11—N2	115.98 (19)	C14—O2—C15	117.8 (2)
C12—C11—C13	126.0 (2)		
			
N3—C2—C3—C4	178.2 (2)	N3—C1—C14—O2	−22.0 (3)
N2—C2—C3—C4	0.3 (2)	C12—C1—C14—O2	157.4 (2)
C2—C3—C4—N1	−0.2 (3)	C3—C4—N1—N2	−0.1 (2)
C2—C3—C4—C5	179.4 (2)	C5—C4—N1—N2	−179.70 (18)
N1—C4—C5—C10	−1.1 (3)	C4—N1—N2—C11	−178.94 (19)
C3—C4—C5—C10	179.3 (2)	C4—N1—N2—C2	0.3 (2)
N1—C4—C5—C6	177.8 (2)	C12—C11—N2—N1	178.77 (19)
C3—C4—C5—C6	−1.8 (3)	C13—C11—N2—N1	0.0 (3)
C10—C5—C6—C7	−0.6 (4)	C12—C11—N2—C2	−0.4 (3)
C4—C5—C6—C7	−179.4 (2)	C13—C11—N2—C2	−179.1 (2)
C5—C6—C7—C8	0.6 (4)	N3—C2—N2—N1	−178.54 (18)
C6—C7—C8—C9	0.3 (4)	C3—C2—N2—N1	−0.4 (2)
C7—C8—C9—C10	−1.2 (4)	N3—C2—N2—C11	0.7 (3)
C8—C9—C10—C5	1.3 (4)	C3—C2—N2—C11	178.88 (19)
C6—C5—C10—C9	−0.4 (4)	C12—C1—N3—C2	−0.4 (3)
C4—C5—C10—C9	178.5 (2)	C14—C1—N3—C2	179.02 (19)
N2—C11—C12—C1	−0.3 (3)	C3—C2—N3—C1	−177.9 (2)
C13—C11—C12—C1	178.3 (2)	N2—C2—N3—C1	−0.3 (3)
N3—C1—C12—C11	0.7 (3)	O1—C14—O2—C15	1.4 (4)
C14—C1—C12—C11	−178.7 (2)	C1—C14—O2—C15	−178.5 (2)
N3—C1—C14—O1	158.1 (3)	C16—C15—O2—C14	147.1 (3)
C12—C1—C14—O1	−22.5 (4)		

### Hydrogen-bond geometry (Å, º)

**Table:**

*D*—H···*A*	*D*—H	H···*A*	*D*···*A*	*D*—H···*A*
C12—H12···O1^i^	0.93	2.36	3.258 (3)	161
C6—H6···N3^ii^	0.93	2.62	3.507 (3)	161

Symmetry codes: (i) −*x*+2, −*y*+1, −*z*; (ii) *x*−1/2, −*y*+3/2, −*z*.
